# Cognitive Health Costs of Poor Housing for Women: Exploring Executive Function and Housing Stress in Urban Slums in India

**DOI:** 10.3390/ijerph21121710

**Published:** 2024-12-23

**Authors:** Uchita Vaid

**Affiliations:** Design Studies Department, University of Wisconsin-Madison, Madison, WI 53706, USA; uvaid@wisc.edu

**Keywords:** housing quality, cognitive health, executive function, slums, informal settlements, India, housing stress, cognitive outcomes, built environment, housing policy

## Abstract

An increasing body of literature has investigated the implications of housing quality on health, confirming the negative consequences of poor housing quality on physical and mental health. Despite this increased focus on the salutogenic impacts of housing, the relationship between housing quality and cognitive health remains understudied. This study examined how the housing quality in urban informal settlements, where living conditions are often substandard, affects women’s cognitive functioning, with a specific focus on executive function (EF) skills. EF is a decision-making system that enables us to make decisions using working memory and attentional control. This study addressed two key questions: (1) Is housing quality associated with EF skills? (2) Does perceived housing stress experienced by women mediate the housing–EF relationship? A standardized observer-based tool assessed housing quality, psychometric instruments measured EF skills, and a 12-item questionnaire evaluated perceived housing stress. Results indicated that better housing quality is positively associated with higher EF skills, with housing stress acting as a mediating factor in this relationship. These findings have important implications for both health and housing policies. Investments in improving housing conditions can yield cognitive health benefits for women, and addressing stress-inducing housing factors could further enhance cognitive outcomes.

## 1. Introduction

With increasing urbanization, urban informal settlements have become a dominant form of housing and now accommodate one-quarter of the world’s population [1]. The prevalence of urban informal settlements is even higher in East, Southeast, Central, and South Asia. Informal settlements or ‘slums’ are characterized by substandard living conditions, including unsafe building structures, poor indoor air quality, crowding, and inadequate access to infrastructural services such as water, sanitation, electricity, and drainage. Studies have well-documented associations between such housing deficiencies and the poor health of slum residents [2].

Living in poor quality housing has been associated with various communicable diseases [3,4], non-communicable diseases including asthma [5] and hypertension [6], higher childhood mortality [7], as well as mental health problems [8,9]; for a review see [10]. In addition to these physical and mental health concerns, it is important to understand the cognitive health impacts of inadequate housing conditions, and additionally, whether housing stress can explain some of the expected relation between housing quality and EF. However, research on the cognitive health impacts of living in informal settlements remains scarce. Two general questions are particularly important for researchers in this area: first, is there a relationship between housing quality and cognitive health? And second, what mechanisms might underlie any observed relationship between inadequate housing and cognitive functioning?

A relationship between inadequate housing and cognitive health can be conceptualized based on foundational research in environmental psychology, neuroscience, and social epidemiology. Studies show that built environmental aspects such as noise, temperature, lighting, access to green spaces, etc., can have consequences for cognitive performance and functioning [11,12,13,14]. Additionally, both chronic and acute stress have well-established connections to cognitive functioning [15,16,17]. In social epidemiology, research on social determinants of cognitive function has investigated various aspects of neighborhoods and built environments, such as neighborhood walkability [18], public transportation use [19], and the indoor built environment [20]. However, much less is known about the relationship between housing quality, housing-related stress, and cognitive functioning—especially executive function (EF) skills. This foundational work in neuroscience and environmental psychology provides a strong rationale for investigating the cognitive consequences of stress associated with living in inadequate housing environments.

Executive function, also called cognitive control or executive control [21], is a set of heterogeneous higher-order cognitive processes which govern human beings’ ability to hold information in mind (i.e., working memory), resist automatic urges (i.e., inhibitory control), and flexibly shift attention (i.e., cognitive flexibility or shifting) [22]. EF can be thought of as the “Airport Control” or decision-making system that enables human beings to make decisions based upon working memory and attentional control [23]. It is a multidimensional construct that encompasses several critical cognitive skills central to the regulation of behavior including working memory, inhibitory control, and attentional control [22]. EF plays a key role in adaptive behavior and top-down regulation of thoughts, emotions, and actions, especially in novel, challenging, or complex situations [24,25,26]. One reason EF could be impacted by housing quality is its sensitivity to stress exposure, and inadequate housing can result in increased stress levels [27]. Both animal studies and human field research support this link between EF and stress [28,29,30].

The current study develops this area of emerging housing and cognitive health research by contributing new data on understanding the EF impacts of living in urban informal settlements. Specifically, it examines whether poorer housing quality is associated with reduced EF skills, and whether perceived housing stress mediates the relationship between housing quality and EF. This study was conducted in urban informal housing settlements as well as redeveloped/renovated urban housing settlements in India. Including both informal and redeveloped settlements allows for variability in housing quality within a study sample that shares a similar socioeconomic status, a known covariate of EF [31].

The following sections review the existing literature on the associations between built environment and EF, as well as the limited literature on the EF skills of residents in informal housing in Low–Middle Income Countries (LMICs).

### 1.1. Built Environment and Executive Function

Research on the relationship between built environment factors and cognitive function has become an increasing focus of scholarly attention in recent years, especially in exploring how our physical surroundings affect our brains—effects that accumulate over time to shape both brain development and cognitive decline. For instance, studies have explored the built environment in neighborhoods and its association with cognitive decline in older adults [32,33]. Built environment factors—such as population density, street connectivity, walkability, public transportation availability, land use diversity, neighborhood resources, green spaces, and overall neighborhood quality—are positively associated with cognitive function in older adults. In contrast, factors like street integration and the distance from residences to main roads have negative associations [34,35].

Specifically, for EF, existing research indicates that household chaos—characterized by housing disorganization and instability—is a significant environmental risk factor that negatively affects EF in children [36,37,38]. However, the associations between household chaos and mothers’ EF are weaker [39,40]. Indoor environmental quality (IEQ) factors, such as noise, also have negative implications for adult EF [41,42], though the evidence is assessed to be weak due to weak study designs, and no significant effect has been found for children’s EF [13,43]. Other IEQ factors, including indoor air quality, lighting, and temperature, have also been linked to EF, with generally negative effects observed under poor IEQ conditions, though some studies found no significant associations. There is more robust evidence for negative associations between indoor air quality and lighting, but evidence is weaker for thermal quality as well as noise [13]. Environmental exposure to metals has also been documented to have a relationship with lower EF skills [44].

Another aspect of the built environment examined in relation to EF is access and exposure to nature or green spaces. Nature exposure can alleviate cognitive overload and reduce stress, which may benefit EF. A systematic review examining the effects of nature interventions on the cognitive functioning of young people (aged 5 to 18) concluded that there is substantial support that nature interventions such as a nature walk, playing in green spaces, gardening, etc., were positively associated with EF [45]. For adults, the relationship between nature and EF has more mixed findings [46,47]. Although there is some evidence linking various built environment factors with EF, the role of housing quality itself as a potential determinant of cognitive health remains underexplored [11].

### 1.2. Executive Function in Informal Housing and LMICs

Most research on EF and its relationship with the built environment has been conducted in high-income countries [46,48], with limited work in LMICs, including India. Furthermore, studies investigating housing and EF, particularly in relation to household chaos, have predominantly focused on populations in North America, Europe, and Australia [36]. This gap in research is critical because evidence from LMICs and vulnerable or historically marginalized populations, such as people living in informal housing, can be particularly valuable, as the potential benefits of such research might be greatest in these settings [49].

A few notable exceptions of EF research in LMICs are reviewed here. A study conducted in an economically vulnerable region of South Africa investigated the association between economic well-being, including a ‘material assets’ assessment of the adequacy of the living environment, with children’s EF [50]. This study found significant associations between ‘material assets’ and all three domains of EF, working memory, attention shifting, and inhibition [50]. In another study in South Africa and Gambia involving children in informal housing, housing materials were assessed as part of housing assets, but no significant associations with EF were found [51]. Studies on children’s EF and the built environment in India have investigated built environment aspects such as exposure to low-level arsenic [52], lead exposure [53], and stimulation levels in homes [54]. However, housing quality in informal settlements in India has not been directly studied in relation to EF.

### 1.3. The Current Study

The current study addresses these gaps by investigating associations between housing quality and EF. It extends the existing EF scholarship conducted in high-income countries by conducting this work in India. Moreover, the study population prioritizes the EF of women residing in urban informal housing, an understudied housing context. This focus on women is crucial because they disproportionately bear the burden of inadequate housing in informal housing in India [55]. This study also addresses current methodological challenges in the housing literature where most work on housing quality and behavior including mental or cognitive outcomes has relied on surveys and self-report measures. This study employs objective housing quality measurement and standardized non-language dependent EF measures to increase methodological rigor, and thus enhance the validity of housing–health claims. Lastly, we investigate whether housing stress acts as a mediating mechanism between housing quality and EF, if any, which can help to understand the housing–EF link better.

## 2. Materials and Methods

### 2.1. Participants

A sample of 154 adult women, aged 19 to 60 years (M = 30.86 ± 8.82), from informal slum and redeveloped public housing settlements in India participated in the study. All participants were from lower socioeconomic backgrounds (mean monthly income = INR 9640, equivalent to approximately USD 115); 97% of participants having less than a high school diploma. Socioeconomic status (SES) was calculated by standardizing and combining income and educational attainment (mean SES index = 0.054 ± 1.55) and was used throughout as a statistical covariate. Table 1 summarizes important sample demographics and independent variables in the study. Housing type (informal slum vs. redeveloped housing) was retained as a covariate in all analyses but did not independently influence the study outcomes. Thus, the data were combined between those living in either slum or redeveloped housing, but the housing type was still included as a statistical covariate in the mediational analysis.

### 2.2. Constructs and Measures

This study examined housing quality and perceived housing stress as independent variables to explore their associations with the dependent variables of executive function (EF), specifically focusing on inhibitory control and working memory.

#### 2.2.1. Housing Quality

Housing quality was assessed using a standardized observer-based instrument, where trained raters evaluated residences across five dimensions using a 124-item rating scale [56]. This tool was adapted for use in informal settlement contexts [27,57] and included five subscales: Cleanliness and Clutter (e.g., presence of dirt on the floor, level of clutter and chaos), Structural Quality (e.g., type and condition of walls, floors, ceilings, presence of peeling paint), Crowding and Privacy (e.g., number of occupants per room, privacy level), Hazards (e.g., exposed toxic substances, presence of safety features like handrails), and Basic Services (e.g., availability of drinking water, sanitation, and electricity). A final score from these subscales was computed to indicate the overall level of housing problems faced by the participants. A higher score on this total score indicated more housing problems. Further details of the scale and its adaptation to informal settlements have been described elsewhere [57]. The Cronbach alpha reliability coefficient for this scale was α = 0.731 in the current sample. As the housing scores were standardized, the range of scores was from −1 to 1, with higher scores indicating more housing issues.

#### 2.2.2. Perceived Housing Stress

The mediator variable, housing stress, was measured using a 12-item scale focusing on the participants’ perceptions of dwelling-related stressors, including disrepair, crowding, unmanageable chores, and ambient noise [58]. Responses were given on a 5-point scale, from “never” to “very often” (e.g., “In the last month, how often have you been upset because of some unexpected problem with the physical condition of your home?”). The total housing stress score was calculated by summing responses across items, with possible scores ranging from 12 to 60, where higher scores indicated more housing-related stress. The Cronbach alpha reliability coefficient for this scale was α = 0.752.

#### 2.2.3. Executive Function

EF, a multidimensional construct involving skills such as working memory, inhibitory control, and cognitive flexibility [21] was assessed using two tasks: the flanker task [59] and the List Sorting Working Memory Test [60].

The flanker task has been widely used to assess selective attention and inhibitory function [61]. In this task, participants were required to identify the direction of a central arrow (the target) flanked by nontarget arrows. Participants pressed the left or right arrow key according to the central target’s direction. In the current study, we used five black arrows against a white background that pointed to the left or right side. Participants were required to focus on the central arrow and to signal the direction of this arrow by pressing a corresponding button on a keyboard with their dominant hand. In different versions of this task, letters have been used instead of arrows [61]. We chose the arrows version due to limited literacy levels in our sample.

**Figure 1:**
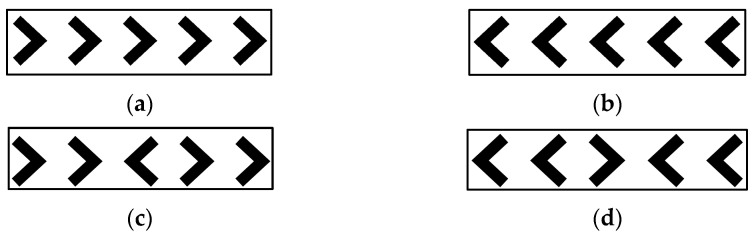
(**a**) Congruent trialswith all arrows pointing right; (**b**) congruent trialswith all arrows pointing left; (**c**) incongruent trialswith central arrow pointing left and others pointing right; (**d**) incongruent trialswith central arrow pointing right and others pointing left.

There were two types of trials including “congruent” or “incongruent” trials. On congruent trials, the direction of the nontarget stimuli matched the target’s direction. Whereas on incongruent trials, the direction of the nontarget stimuli pointed in the opposite direction of the target (Figure 1). Therefore, choosing a correct response is more challenging on incongruent trials in comparison to congruent trials. Participants were instructed to respond to the target stimuli as quickly and as accurately as possible.

The participants were presented with a welcome screen and task instructions which were explained by the experimenter in the local language. After ensuring that participants understood the task, a practice session of 20 trials with feedback was provided, and participants needed at least 70% accuracy to proceed to the main trials. If accuracy was sufficient, participants then completed three main sets of 25 trials each. Both accuracy and choice reaction time were measured on each trial. The difference between the choice reaction time for congruent trials and incongruent trials was computed as a measure of inhibitory control [62].

The List Sorting Working Memory Test was used to measure working memory [60]. This is a sequencing task requiring participants to remember, sort information, and sequence it. Participants were presented with a series of names (e.g., animals or a combination of animals and food items) and were instructed to recall and sequence them in order of size, from smallest to largest. Two lists were presented: the first list included only animals, and the second included a mix of animals and foods. The number of objects in a series increased on successive items, thereby taxing the working memory system with longer sequences. Working memory was scored based on the number of items correctly recalled in the specified order, with a final score representing the average across the two lists. Scores ranged from 1 to 7.

## 3. Results

As shown in Table 2, significant associations were observed among housing problems, housing stress, flanker reaction time, and working memory. However, flanker accuracy did not significantly correlate with any other variables.

### 3.1. Housing Problems and EF

To analyze the relationship between housing problems and EF, OLS regression models were used that included housing problems as the independent variable and flanker accuracy score, flanker reaction time, and working memory score as the dependent variables in respective regression models. Participants’ age, SES, and housing type (slum/redeveloped settlement, with slum as the reference category) were included as covariates in all three regression models.

Table 3 summarizes the regression analysis results for each dependent variable. Housing problems did not significantly predict the flanker accuracy score (*p* = 0.868). However, age was a significant predictor (*β*(SE) = −0.003(0.001), *p* = 0.028), indicating that the flanker accuracy score decreased with age. Housing problems significantly predicted the flanker reaction time (*β*(SE) = 182.249(22.266), *p* < 0.001); as housing problems increased, the flanker reaction time also increased significantly. A higher flanker reaction time indicated worse EF. Housing type (*β*(SE) = −43.005(15.130), *p* = 0.005) and age (*β*(SE) = −1.676(0.756), *p* = 0.029) were also significant in predicting the flanker reaction time. Residents in informal slum settlements reported significantly higher flanker reaction times (M = 116.49), reflecting lower EF compared to residents in redeveloped housing (M = 54.72). Additionally, flanker reaction time decreased with age.

For working memory, housing problems were a significant predictor (*β*(SE) = −0.734(0.226), *p* = 0.001), with higher levels of housing problems associated with lower working memory scores. Housing type (*β*(SE) = 0.421(0.156), *p* = 0.008) and SES (*β*(SE) = 0.131(0.053), *p* = 0.015) also significantly predicted working memory scores. Participants from slum settlements had lower working memory scores (M = 2.55) than those in redeveloped housing (M = 2.99), and working memory improved with increasing SES.

See Supplementary Materials for the results of the regression models without the inclusion of housing type as a covariate. The exclusion of housing type did not impact the regression results significantly as housing problems were still a significant predictor for flanker reaction time as well as working memory (Table S1 in Supplementary File). Additionally, supplementary analyses were conducted to explore whether each of the subscales of housing quality has significant relationships with EF. Due to the exploratory nature of these analyses, they have been included in the Supplementary File (Tables S2 and S3 in Supplementary File).

### 3.2. Housing Problems, Housing Stress, and EF

To assess the mediating role of housing stress, mediation analyses with 5000 bootstrap samples were conducted using PROCESS (model 4) [63] were conducted. All mediation models included age, SES, and housing type as covariates.

**Figure 2:**
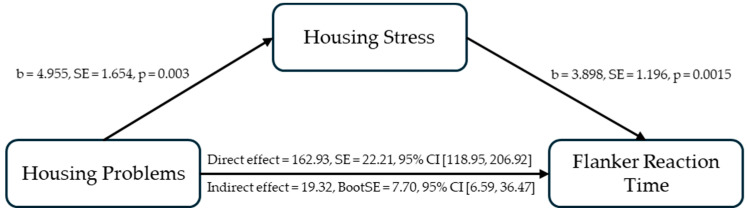
Housing stress as a mediator in explaining the relationship between housing problems and flanker reaction time.

The mediation model predicting flanker accuracy did not show any significant direct effect of housing problems [95%CI = [−0.084, 0.078]] or indirect effect of housing stress [95%CI = [−0.026, 0.019]. For flanker reaction time, when housing stress was entered as a mediator, greater housing problems were significantly associated with higher housing stress, b = 4.955, SE = 1.654, t = 2.995, *p* = 0.003, 95%CI = [1.679, 8.231]. Higher housing stress also significantly predicted greater flanker reaction times, b = 3.898, SE = 1.196, t = 3.259, *p* = 0.0015, 95%CI = [1.529, 6.267]. When accounting for housing stress, the association between housing problems and flanker reaction time weakened (b = 162.932, SE = 22.209, t = 7.336, *p* < 0.001, 95% CI [118.945, 206.920]) from its previous level (b = 182.249, SE = 22.266, t = 8.185, *p* < 0.001, 95% CI [138.153, 226.346]). Crucially, the indirect effect through housing stress significantly explained a part of the relationship between housing problems and flanker reaction time (indirect effect = 19.317, BootSE = 7.703, 95% CI [6.593, 36.465]) (see Figure 2). The mediating role of housing stress was significant in partially explaining the positive relationship between housing problems and flanker reaction time.

**Figure 3:**
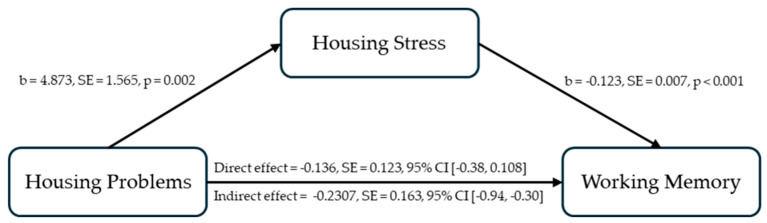
Housing stress as a mediator in explaining the relationship between housing problems and working memory.

Similarly, for working memory, higher housing stress was associated with lower working memory scores, b = −0.123, SE = 0.007, t = −18.210, *p* < 0.001, 95%CI = [−0.136, −109]. After adding housing stress, the relationship between housing problems and working memory became non-significant, b = −0.136, SE = 0.123, t = −1.103, *p* = 0.272, 95% CI [−0.380, 0.108] (from a significant relationship, b = −0.734, SE = 0.226, t = −3.253, *p* = 0.0015, 95% CI [−1.180, −0.287]). The indirect pathway through housing stress significantly explained the relationship between housing problems and working memory, indirect effect = −0.2307, BootSE = 0.163, 95% CI [−0.943, −0.301] (see Figure 3). The mediating role of housing stress was significant in completely explaining the negative relationship between housing problems and working memory.

## 4. Discussion

The present study investigated the relationship between housing quality and executive function (EF) skills in women residing in urban informal housing communities in India. Additionally, it examined whether housing stress could explain the link between housing quality and EF. To capture a range of housing conditions, we included both women living in urban slum housing and those in redeveloped or improved housing, thus allowing for a comparison of substandard and better housing among women of similar socioeconomic status. As hypothesized, housing quality predicted EF scores, specifically as reflected in flanker reaction time and working memory scores, but not in flanker accuracy. Further, housing stress mediated the relationship between housing quality and EF, with consistent findings even when controlling for socioeconomic status (SES), age, and housing type.

### 4.1. Housing Quality, Housing Stress, and Inhibitory Control

Inhibitory control is a critical executive function that enables individuals to delay impulsive actions and manage automatic responses [24]. This ability to suppress habitual or prepotent responses helps facilitate appropriate actions, which is essential for adapting to complex daily demands and shifting environments [64]. Findings from this study showed that women living in poorer housing conditions had greater interference effects in reaction times on the flanker task, indicating weaker inhibitory control compared to women in higher-quality housing. The increased interference effect, measured by the difference in reaction time between incongruent (Figure 1c,d) and congruent (Figure 1a,b) trails, has been suggested to reflect impaired inhibitory control [62]. Previous work on built environment aspects such as indoor environmental quality, noise exposure, and exposure to hazardous elements have found similar associations with EF [11,12,13,14]. Our findings extend this understanding by highlighting housing quality as another environmental factor with significant connections to inhibitory control. One potential explanation for this relationship is the psychological stress caused by substandard housing conditions (e.g., crowding, pests, temporary materials, and limited access to water or sanitation) [55,56]. To further investigate, we tested a mediation model to assess whether housing stress could explain the link between housing quality and EF, which is discussed later in this section.

Age was also a significant predictor of inhibitory control, with older women demonstrating lower inhibitory control than younger women. Prior research comparing inhibitory control across age groups has consistently shown that reaction times improve with age in children [65,66] but tend to slow in older adults [67,68]. Neuroimaging studies suggest that age-related declines in inhibitory control may stem from altered activation in the frontal and parietal lobes and the prefrontal cortex [69]. Additionally, behavioral and neuropsychological studies have demonstrated that older adults tend to show greater variability in response times compared to younger adults [70]. Future studies on housing environments can investigate the relationships between housing aspects and prefrontal cortex activations across the life span, to understand the mediating mechanisms of relationships between age, housing, and inhibitory control.

Housing type also significantly predicted inhibitory control, with women living in informal slum housing displaying lower inhibitory control compared to those in redeveloped housing, after accounting for age and housing quality. This finding indicates that factors other than housing quality differences between the two housing types could have associations with inhibitory control. Future research could explore social stressors specific to these housing types such as a lack of social cohesion [71], as social stress has been linked to inhibitory control outcomes [72].

Finally, housing stress was found to partially mediate the association between housing quality and inhibitory control, which aligns with previous findings linking elevated stress to reduced inhibitory control [73,74,75]. The results of this study contribute to the literature on stress and EF, extending beyond existing evidence for acute and chronic stressors such as economic and relational stress, by offering new evidence regarding the role of housing stress in this relationship.

### 4.2. Housing Quality, Housing Stress, and Working Memory

Working memory enables individuals to temporarily remember information while competitively processing information [76]. A crucial function of the working memory system is distinguishing between relevant and irrelevant information while maintaining task goals, often in the face of competing or distracting information [77]. Impaired working memory can be a hindrance in everyday situations, for it is often necessary to attend to important information while directing attention away from less relevant information. This study found that housing quality, housing type, and SES significantly predicted women’s working memory.

Women living in lower-quality housing had worse working memory than women in better-quality housing. This relationship was fully mediated by housing stress, where the association between housing quality and working memory became insignificant when housing stress was accounted for. This finding indicates that the stresses associated with living in substandard-quality housing tax the cognitive capacities and thus have impacts on working memory, an important aspect of cognitive functioning. The existing research indicates that repeated exposure to environmental stressors such as noise, crowding, etc., heightens the “costs of coping” and coping fatigue [78]. Placing the body’s coping system in overdrive for extended periods of time can impact cognitive functioning [79].

Women with lower SES had worse working memory than women with higher SES. This finding corroborates existing work on poverty and cognitive outcomes, especially in children [80]. A quasi-experimental study with adults also found SES differences associated with cognitive functioning [73]. Age was not significantly associated with working memory in our findings. This contrasts existing work on cognitive decline with age, specifically related to working memory [81,82]. A possible explanation for this finding could be that the measure used in this study may not be as sensitive to age-related changes in the study population. Further research across the life span with women living in urban informal housing is needed to identify sensitivity of the measures.

Moreover, women living in informal slum housing were found to have worse working memory in comparison to women in redeveloped housing. Existing studies have found significant effects of built environment aspects such as urbanity, residential density, walkability, physical disorder, and green spaces on memory and cognition [83]. Since informal slum housing has been documented to have distinct built environment ecology [84], more research is needed on relevant built environment factors for cognition in these housing communities.

### 4.3. Study Limitations

Although the present findings are potentially quite important given the essential role of EF in physical health and psychological well-being, the data are all cross-sectional, limiting causal inferences at this stage of research. Given the homogeneous sample and statistical controls for SES and age, it is unlikely that poverty or a related personal variable is responsible for the observed findings. However, to strengthen these conclusions, especially the mediation results, an ideal approach would involve investigating participants randomly assigned to varying levels of housing quality or, more feasibly, tracking individuals over time as their housing conditions change, alongside longitudinal assessments of EF. Such a prospective longitudinal design would allow for a more rigorous examination of housing stress as a mediator, assuming its role as a temporal link between housing quality and EF. True mediation cannot be inferred from the current cross-sectional data for which longitudinal data are necessary. Finally, we note that this study used a convenience sample of volunteer participants living in urban informal housing, so a self-selection bias cannot be ruled out.

## 5. Conclusions

Many studies have shown adverse health sequelae of residing in substandard housing. There is growing evidence that people living in informal housing, who face cumulative disadvantages that are exacerbated by poor-quality housing, have exacerbated health impacts [85,86,87]. The present study builds upon and extends this large body of housing and health work in three ways. First, it has shown for the first time that poor quality housing may also be capable of damaging executive function, an important aspect of healthy human functioning. We reason that since living in a poor-quality house is likely stressful, that the chronic stress associated therewith would be capable of disrupting EF. Also, it provides evidence that housing related stress also has detrimental EF impacts and explains the housing–EF relationship. This extension strengthens the existing housing and health evidence.

Second, this study’s findings are valuable as they move beyond the common reliance on self-reported measures in housing and health research [88]. By using trained raters to assess housing quality and standardized EF measures for cognitive health, this study provides methodological rigor, enhancing the validity of housing–health claims. Third, this study addresses housing quality in an LMIC, in India, where little research exists on housing and cognitive health among low-SES women. Given that more people live in LMICs in worse housing than the samples used in extant research on housing and health, expanding housing–health research to diverse samples and housing types is essential. Further research should also consider how housing quality interacts with other environmental (e.g., neighborhood quality) and social risk factors (e.g., family crisis) that often compound challenges in urban informal housing. These findings offer critical insights for housing and health policies aimed at supporting cognitive health in vulnerable populations.

## Figures and Tables

**Table 1:** Summary of basic descriptive statistics for the variables included in the model.

	N (%)/M (SD)
Housing Type Informal slum settlement Redeveloped settlement	154 (100%) 79 (51.3%) 75 (48.7%)
Age (in years)	30.86 (8.82)
Employment status Employed full time Employed part time Unemployed	154 (100%) 18 (11.7%) 44 (28.6%) 92 (59.7%)
Household income (in INR/month)	9640.15 (3265.78)
Education (grade)	3.20 (3.82)
Socioeconomic status	0.054 (1.553)
Housing problems	0.0009 (0.337)
Housing stress	37.221 (5.611)

Note: Mean (M) and standard deviation (SD) values are shown for age, household income, education, housing problems, and housing stress. All remaining values indicate N = sample size (percentages).

**Table 2:** The inter-correlations among key variables.

	Housing Problems	Housing Stress	Flanker Accuracy	Flanker Reaction Time	Working Memory
Housing Problems	1	0.364 **	−0.091	0.657 **	−0.405 **
Housing Stress	0.364 **	1	−0.081	0.473 **	−0.854 **
Flanker Accuracy	−0.091	−0.081	1	−0.072	0.119
Flanker Reaction Time	0.657 **	0.473 **	−0.072	1	−0.468 **
Working Memory	−0.405 **	−0.854 **	0.119	−0.468 **	1

Note: ** *p* < 0.001.

**Table 3:** Results of linear regression models for predicting flanker accuracy score, flanker reaction time, and working memory score.

	Flanker Accuracy Score		Flanker Reaction Time		Working Memory	
	B(SE), *p*	*t*	B(SE), *p*	t	B(SE), *p*	t
Constant	0.972 (0.061) ***	15.813	199.867 (34.815) ***	5.741	2.165 (0.364) ***	5.953
Housing Problems	−0.007 (0.039)	−0.166	182.249 (22.266) ***	8.185	−0.734 (0.226) **	−3.253
Housing Type	−0.002 (0.027)	−0.085	−43.005 (15.130) **	−2.842	0.421 (0.156) **	2.697
Age	−0.003 (0.001) *	−2.223	−1.676 (0.756) *	−2.216	−0.001 (0.008)	−0.143
SES	0.015 (0.009)	1.705	−5.727 (5.028)	−1.139	0.131 (0.053) *	2.469

Note: B = unstandardized regression coefficient; SE = standard error; t = t-statistic; flanker accuracy model: R^2^ = 0.101 (adjusted R^2^ = 0.071), F(4, 117) = 3.299, *p* = 0.013; flanker reaction time model: R^2^ = 0.505 (adjusted R^2^ = 0.488), F(4, 117) = 29.853, *p* < 0.001; working memory model: R^2^ = 0.213 (adjusted R^2^ = 0.188), F(4, 127) = 8.60, *p* < 0.001. * *p* < 0.05; ** *p* < 0.01; *** *p* < 0.001.

## Data Availability

The dataset presented in this article are not readily available because the data are part of an ongoing study. Requests to access the datasets should be directed to Uchita Vaid at uvaid@wisc.edu.

## Supplementary Materials

The following supporting information can be downloaded at: https://www.mdpi.com/article/10.3390/ijerph21121710/s1, Table S1. Results of linear regression models for predicting flanker accuracy score, flanker reaction time, and working memory score without housing type as a covariate; Table S2. Results of linear regression models for predicting flanker accuracy score, flanker reaction time, and working memory score with disaggregated housing quality subscales; Table S3. Results of linear regression model for predicting housing stress with disaggregated housing quality subscales.

## References

[1] UN-Habitat World Cities Report 2020: The Value of Sustainable Urbanization. https://unhabitat.org/sites/default/files/2020/11/world_cities_report_2020_abridged_version.pdf.

[2] Ezeh A., Oyebode O., Satterthwaite D., Chen Y.F., Ndugwa R., Sartori J., Mberu B., Melendez-Torres G.J., Haregu T., Watson S.I. (2017). The history, geography, and sociology of slums and the health problems of people who live in slums. Lancet.

[3] Baker E., Beer A., Lester L., Pevalin D., Whitehead C., Bentley R. (2017). Is housing a health insult?. Int. J. Environ. Res. Public Health.

[4] Taylor L. (2018). Housing and health: An overview of the literature. Health Affairs Health Policy Brief.

[5] Wimalasena N., Chang-Richards A., Wang K., Dirks K. (2021). Housing risk factors associated with respiratory disease: A systematic review. Int. J. Environ. Res. Public Health.

[6] Jacobs D.E., Wilson J., Dixon S.L., Smith J., Evens A. (2009). The relationship of housing and population health: A 30-year retrospective analysis. Environ. Health Perspect..

[7] Tusting L.S., Gething P.W., Gibson H.S., Greenwood B., Knudsen J., Lindsay S.W., Bhatt S. (2020). Housing and child health in sub-Saharan Africa: A cross-sectional analysis. PLoS Med..

[8] Subbaraman R., Nolan L., Shitole T., Sawant K., Shitole S., Sood K., Nanarkar M., Ghannam J., Bloom D.E., Patil-Deshmukh A. (2014). The psychological toll of slum living—An assessment of mental health, disability, and slum-related adversities in Mumbai, India. Lancet Glob. Health.

[9] Singh A., Daniel L., Baker E., Bentley R. (2019). Housing disadvantage and poor mental health: A systematic review. Am. J. Prev. Med..

[10] Howden-Chapman P., Bennett J., Edwards R., Jacobs D., Nathan K., Ormandy D. (2023). Review of the impact of housing quality on inequalities in health and well-being. Annu. Rev. Public Health.

[11] Campos-Gil J.A., Ortega-Andeane P., Vargas D. (2020). Children’s microsystems and their relationship to stress and executive functioning. Front. Psychol..

[12] Hygge S., Knez I. (2001). Effects of noise, heat and indoor lighting on cognitive performance and self-reported affect. J. Environ. Psychol..

[13] Wang C., Zhang F., Wang J., Doyle K., Hancock A., Mak M., Liu S. (2021). How indoor environmental quality affects occupants’ cognitive functions: A systematic review. Build. Environ..

[14] Reyes-Riveros R., Altamirano A., De La Barrera F., Rozas-Vásquez D., Vieli L., Meli P. (2021). Linking public urban green spaces and human well-being: A systematic review. Urban For. Urban Green..

[15] McEwen B.S. (2007). Physiology and neurobiology of stress and adaptation: Central role of the brain. Physiol. Rev..

[16] Shields S., Sazma A., Yonelinas P. (2016). The effects of acute stress on core executive functions: A meta-analysis and comparison with cortisol. Neurosci. Biobehav. Rev..

[17] Law R., Clow A. (2020). Stress, the cortisol awakening response and cognitive function. Int. Rev. Neurobiol..

[18] Ng P., Nyunt Z., Shuvo K., Eng Y., Yap B., Hee M., Chan S.P., Scherer S. (2018). The neighborhood built environment and cognitive function of older persons: Results from the Singapore longitudinal ageing study. Gerontology.

[19] Reinhard E., Carrino L., Courtin E., van Lenthe F.J., Avendano M. (2019). Public transportation use and cognitive function in older age: A quasi experimental evaluation of the free bus pass policy in the United Kingdom. Am. J. Epidemiol..

[20] Leung Y., Wang C., Famakin O. (2021). Integrated model for indoor built environment and cognitive functional ability of older residents with dementia in care and attention homes. Build. Environ..

[21] Diamond A. (2013). Executive functions. Annu. Rev. Psychol..

[22] Miyake A., Friedman P., Emerson J., Witzki H., Howerter A., Wager D. (2000). The unity and diversity of executive functions and their contributions to complex “frontal lobe” tasks: A latent variable analysis. Cogn. Psychol..

[23] Shonkoff J.P. (2012). Leveraging the biology of adversity to address the roots of disparities in health and development. Proc. Natl. Acad. Sci. USA.

[24] Hofmann W., Schmeichel J., Baddeley D. (2012). Executive functions and self-regulation. Trends Cogn. Sci..

[25] Jurado B., Rosselli M. (2007). The elusive nature of executive functions: A review of our current understanding. Neuropsychol. Rev..

[26] Roebers C.M. (2017). Executive function and metacognition: Towards a unifying framework of cognitive self-regulation. Dev. Rev..

[27] Vaid U. (2023). Physical and mental health impacts of housing improvement: A quasi-experimental evaluation of in-situ slum redevelopment in India. J. Environ. Psychol..

[28] Blair C. (2010). Stress and the development of self-regulation in context. Child Dev. Perspect..

[29] Juster P., McEwen S., Lupien J. (2010). Allostatic biomarkers of chronic stress and impact on health and cognition. Neurosci. Biobehav. Rev..

[30] Repetti L., Taylor E., Seeman E. (2002). Risky families: Family social environments and the mental and physical health of offspring. Psychol. Bull..

[31] Lawson G.M., Hook C.J., Farah M.J. (2018). A meta-analysis of the relationship between socioeconomic status and executive function performance among children. Dev. Sci..

[32] Wu T., Prina M., Jones A., Matthews E., Brayne C. (2017). The built environment and cognitive disorders: Results from the cognitive function and ageing study II. Am. J. Prev. Med..

[33] Besser M., Chang C., Hirsch A., Rodriguez A., Renne J., Rapp R., Fitzpatrick A.L., Heckbert S.R., Kaufman J.D., Hughes M. (2021). Longitudinal associations between the neighborhood built environment and cognition in US older adults: The multi-ethnic study of atherosclerosis. Int. J. Environ. Res. Public Health.

[34] Song Y., Liu Y., Bai X., Yu H. (2024). Effects of neighborhood built environment on cognitive function in older adults: A systematic review. BMC Geriatr..

[35] Besser M., McDonald C., Song Y., Kukull A., Rodriguez A. (2017). Neighborhood environment and cognition in older adults: A systematic review. Am. J. Prev. Med..

[36] Andrews K., Atkinson L., Harris M., Gonzalez A. (2021). Examining the effects of household chaos on child executive functions: A meta-analysis. Psychol. Bull..

[37] Wachs T., Evans G., Evans G., Wachs T. (2010). Chaos in context. Chaos and Its Influence on Children’s Development: An Ecological Perspective.

[38] Andrews K., Dunn R., Prime H., Duku E., Atkinson L., Tiwari A., Gonzalez A. (2021). Effects of household chaos and parental responsiveness on child executive functions: A novel, multi-method approach. BMC Psychol..

[39] Deater-Deckard K., Chen N., Wang Z., Bell M.A. (2012). Socioeconomic risk moderates the link between household chaos and maternal executive function. J. Fam. Psychol..

[40] Marsh S., Dobson R., Maddison R. (2020). The relationship between household chaos and child, parent, and family outcomes: A systematic scoping review. BMC Public Health.

[41] Clark C., Paunovic K. (2018). WHO environmental noise guidelines for the European region: A systematic review on environmental noise and cognition. Int. J. Environ. Res. Public Health.

[42] Thompson R., Smith R.B., Karim Y.B., Shen C., Drummond K., Teng C., Toledano M.B. (2022). Noise pollution and human cognition: An updated systematic review and meta-analysis of recent evidence. Environ. Int..

[43] Belojevic G., Evans G., Paunovic K., Jakovljevic B. (2012). Traffic noise and executive functioning in urban primary school children: The moderating role of gender. J. Environ. Psychol..

[44] Rafiee A., Delgado-Saborit J.M., Sly P.D., Quemerais B., Hashemi F., Akbari S., Hoseini M. (2020). Environmental chronic exposure to metals and effects on attention and executive function in the general population. Sci. Total Environ..

[45] Vella-Brodrick D.A., Gilowska K. (2022). Effects of nature (greenspace) on cognitive functioning in school children and adolescents: A systematic review. Educ. Psychol. Rev..

[46] Jimenez M.P., DeVille N.V., Elliott E.G., Schiff J.E., Wilt G.E., Hart J.E., James P. (2021). Associations between nature exposure and health: A review of the evidence. Int. J. Environ. Res. Public Health.

[47] Ricciardi E., Spano G., Lopez A., Tinella L., Clemente C., Elia G., Dadvand P., Sanesi G., Bosco A., Caffò A.O. (2022). Long-term exposure to greenspace and cognitive function during the lifespan: A systematic review. Int. J. Environ. Res. Public Health.

[48] Bradley R.H., Corwyn R.F. (2002). Socioeconomic status and child development. Annu. Rev. Psychol..

[49] Ferguson K.T., Cassells R.C., MacAllister J.W., Evans G.W. (2013). The physical environment and child development: An international review. Int. J. Psychol..

[50] Turbeville A., Aber J.L., Weinberg S.L., Richter L., van Heerden A. (2019). The relationship between multidimensional economic well-being and children’s mental health, physical health, and executive function development in South Africa. Dev. Sci..

[51] Milosavljevic B., Cook C.J., Fadera T., Ghillia G., Howard S.J., Makaula H., Mbye E., McCann S., Merkley R., Mshudulu M. (2023). Executive functioning skills and their environmental predictors among pre-school aged children in South Africa and The Gambia. Dev. Sci..

[52] Vaidya N., Holla B., Heron J., Sharma E., Zhang Y., Fernandes G., Iyengar U., Spiers A., Yadav A., Das S. (2023). Neurocognitive analysis of low-level arsenic exposure and executive function mediated by brain anomalies among children, adolescents, and young adults in India. JAMA Netw. Open.

[53] Roy A., Bellinger D., Hu H., Schwartz J., Ettinger A.S., Wright R.O., Bouchard M., Palaniappan K., Balakrishnan K. (2009). Lead exposure and behavior among young children in Chennai, India. Environ. Health Perspect..

[54] Obradović J., Finch J.E., Portilla X.A., Rasheed M.A., Tirado-Strayer N., Yousafzai A. (2019). Early executive functioning in a global context: Developmental continuity and family protective factors. Dev. Sci..

[55] Parikh P., Fu K., Parikh H., McRobie A., George G. (2015). Infrastructure provision, gender, and poverty in Indian slums. World Dev..

[56] Evans G., Wells N., Chan H., Saltzman H. (2000). Housing quality and mental health. J. Consult. Clin. Psychol..

[57] Vaid U. (2021). Delivering the promise of ‘better homes’?: Assessing housing quality impacts of slum redevelopment in India. Cities.

[58] Campagna G. (2016). Linking crowding, housing inadequacy, and perceived housing stress. J. Environ. Psychol..

[59] Eriksen B.A., Eriksen C.W. (1974). Effects of noise letters upon the identification of a target letter in a nonsearch task. Percept. Psychophys..

[60] Tulsky D., Carlozzi N., Chevalier N., Espy K., Beaumont J., Mungas D. (2013). NIH toolbox cognition battery (CB): Measuring working memory. Monogr. Soc. Res. Child Dev..

[61] Ridderinkhof K.R., Wylie S.A., van den Wildenberg W.P., Bashore T.R., van der Molen M.W. (2021). The arrow of time: Advancing insights into action control from the arrow version of the Eriksen flanker task. Atten. Percept. Psychophys..

[62] Rueda M., Fan J., McCandliss B., Halparin J., Gruber D., Lercari L., Posner M. (2004). Development of attentional networks in childhood. Neuropsychologia.

[63] Hayes A.F. (2018). Introduction to Mediation, Moderation, and Conditional Process Analysis: A Regression-Based Approach.

[64] Horváth K., Nemeth D., Janacsek K. (2022). Inhibitory control hinders habit change. Sci. Rep..

[65] Williams B.R., Ponesse J.S., Schachar R.J., Logan G.D., Tannock R. (1999). Development of inhibitory control across the life span. Dev. Psychol..

[66] Macdonald J.A., Beauchamp M.H., Crigan J.A., Anderson P.J. (2014). Age-related differences in inhibitory control in the early school years. Child Neuropsychol..

[67] Christ S.E., White D.A., Mandernach T., Keys B.A. (2001). Inhibitory control across the life span. Dev. Neuropsychol..

[68] McAuley T., Yap M., Christ S.E., White D.A. (2006). Revisiting inhibitory control across the life span: Insights from the ex-Gaussian distribution. Dev. Neuropsychol..

[69] Nielson K.A., Langenecker S.A., Garavan H. (2002). Differences in the functional neuroanatomy of inhibitory control across the adult life span. Psychol. Aging.

[70] Cowell R.A., Paitel E.R., Peters S. (2020). The context of caution: An examination of age, social context, and sex on measures of inhibitory control and risky decision-making. Int. J. Aging Hum. Dev..

[71] Vaid U. (2024). Shifting landscapes: Women’s experiences of socio-cultural changes from in-situ redevelopment in Ahmedabad. J. Environ. Psychol..

[72] Sánchez-Salvador L., Prados-Pardo Á., Martín-González E., Olmedo-Córdoba M., Mora S., Moreno M. (2021). The Role of Social Stress in the Development of Inhibitory Control Deficit: A Systematic Review in Preclinical Models. Int. J. Environ. Res. Public Health.

[73] Mani A., Mullainathan S., Shafir E., Zhao J. (2013). Poverty impedes cognitive function. Science.

[74] Mika A., Mazur G.J., Hoffman A.N., Talboom J.S., Bimonte-Nelson H.A., Sanabria F., Conrad C.D. (2012). Chronic stress impairs prefrontal cortex-dependent response inhibition and spatial working memory. Behav. Neurosci..

[75] Roos L.E., Knight E.L., Beauchamp K.G., Berkman E.T., Faraday K., Hyslop K., Fisher P.A. (2017). Acute stress impairs inhibitory control based on individual differences in parasympathetic nervous system activity. Biol. Psychol..

[76] Miyake A., Shah P. (1999). Models of Working Memory.

[77] Engle R.W., Tuholski S.W., Laughlin J.E., Conway A.R. (1999). Working memory, short-term memory, and general fluid intelligence: A latent-variable approach. J. Exp. Psychol. Gen..

[78] Evans G., Cohen S., Spielberger C. (2004). Environmental Stress. Encyclopedia of Applied Psychology.

[79] Cohen S., Evans G.W., Stokols D., Krantz D.S. (1986). Behavior, Health, and Environmental Stress.

[80] Duncan G.J., Magnuson K. (2012). Socioeconomic status and cognitive functioning: Moving from correlation to causation. Wiley Interdiscip. Rev. Cogn. Sci..

[81] Hayes M.G., Kelly A.J., Smith A.D. (2013). Working memory and the strategic control of attention in older and younger adults. J. Gerontol. Ser. B Psychol. Sci. Soc. Sci..

[82] Brockmole J.R., Logie R.H. (2013). Age-related change in visual working memory: A study of 55,753 participants aged 8–75. Front. Psychol..

[83] Chen X., Lee C., Huang H. (2022). Neighborhood built environment associated with cognition and dementia risk among older adults: A systematic literature review. Soc. Sci. Med..

[84] Dovey K., van Oostrum M., Chatterjee I., Shafique T. (2020). Towards a morphogenesis of informal settlements. Habitat Int..

[85] Zerbo A., Delgado R.C., González P.A. (2020). Vulnerability and everyday health risks of urban informal settlements in Sub-Saharan Africa. Glob. Health J..

[86] Anand A., Phuleria H.C. (2022). Assessment of indoor air quality and housing, household and health characteristics in densely populated urban slums. Environ. Dev. Sustain..

[87] Weimann A., Oni T. (2019). A systematised review of the health impact of urban informal settlements and implications for upgrading interventions in South Africa, a rapidly urbanising middle-income country. Int. J. Environ. Res. Public Health.

[88] Alidoust S., Huang W. (2023). A decade of research on housing and health: A systematic literature review. Rev. Environ. Health.

