# Supplementary material for: Cognitive Health Costs of Poor Housing for Women: Exploring Executive Function and Housing Stress in Urban Slums in India
Source: Int J Environ Res Public Health. 2024 Dec 23;21(12):1710. doi: 10.3390/ijerph21121710 (PMC11727654; doi:10.3390/ijerph21121710)
Supplement: Supplementary file 1 [file ijerph-21-01710-s001.zip › ijerph-3333312-supplementary.pdf]

Supplementary Analysis

Table S1. Results of linear regression models for predicting flanker accuracy score, flanker reaction time, and working memory score without housing type as a covariate.

	Flanker Accuracy Score		Flanker Reaction Time		Working Memory	
	**B(SE), p**	***t***	**B(SE), p**	**t**	**B(SE), p**	**t**
Constant	0.968 (0.042)***	22.841	128.48(24.82)***	5.741	2.877(0.256)***	5.953
Housing Problems	-0.005(0.036)	-.145	206.484(21.18)***	9.751	-0.952(0.216)***	-4.418
Age	-0.003(0.001)*	-2.234	-1.480(0.776)	-1.909	-0.003(0.008)	-0.429
SES	0.016(0.008)*	2.047	1.620(4.44)	.365	0.056 (0.046)	1.208

Note: B = unstandardized regression coefficient; SE = standard error; t = t-statistic; flanker accuracy model: $R^2 = 0.101$ (adjusted $R^2 = 0.078$), $F(3, 118) = 4.434$, $p = 0.005$; flanker reaction time model: $R^2 = 0.471$(adjusted $R^2 = 0.457$), $F(3, 118) = 35.011$, $p < 0.001$; working memory model: $R^2 = 0.168$ (adjusted $R^2 = 0.149$), $F(3, 128) = 8.62$, $p < 0.001$.
* p < .05; **p < .01; ***p < .001

Results from Tables S2 and S3 should be interpreted within the context of a few caveats. First, the disaggregation of composite scales is not advisable since these are standard scales designed to be used as overall measures and disaggregating them can reduce the overall robustness. The housing quality scale is disaggregated here only for exploratory purposes. And second, conducting multiple inferential tests raises the problem of overall experimenter error rate (type 1 error). Thus, the following analyses are more exploratory instead of being inferential.

Table S2. Results of linear regression models for predicting flanker accuracy score, flanker reaction time, and working memory score with disaggregated housing quality subscales.

	Flanker Accuracy Score		Flanker Reaction Time		Working Memory	
	**B(SE), p**	***t***	**B(SE), p**	**t**	**B(SE), p**	**t**
Constant	.971 (.044)***	21.911	126.625 (25.64)***	4.938	2.876 (.245)***	11.749
Age	-0.003(0.001)	-2.202	-1.387(0.804)	-1.726	-0.004(0.008)	-0.527
SES	0.016(0.008)	1.874	-1.598(4.797)	-0.333	0.097(0.046)*	2.106
Cleanliness and Clutter	-0.009(.025)	-0.336	17.989(14.671)	1.226	-0.073(0.14)	-0.521
Basic Services	-0.010(0.041)	-0.245	64.972(23.991)**	2.708	-1.038(0.231)***	-4.503
Hazards	0.034(0.043)	0.775	36.473(25.077)	1.454	0.225(0.232)	0.971
Structural Quality	-0.017(.03)	-0.574	55.063(17.432)**	3.159	-0.385(0.162)*	-2.381
Crowding	0.026(0.039)	0.656	38.665(22.557)	1.714	0.263(0.22)	1.193

Note: B = unstandardized regression coefficient; SE = standard error; t = t-statistic; flanker accuracy model: $R^2 = 0.112$ (adjusted $R^2 = .057$), $F(7, 114) = 2.045$, $p = .055$; flanker reaction time model: $R^2 = 0.490$(adjusted $R^2 = .458$), $F(7, 114) = 15.616$, $p < 0.001$; working memory model: $R^2 = 0.311$ (adjusted $R^2 = .272$), $F(7, 124) = 7.985$, $p < 0.001$.
* p < .05; **p < .01; ***p < .001

Table S3. Results of linear regression model for predicting housing stress with disaggregated housing quality subscales.

	Housing Stress	
	**B(SE), p**	***t***
Constant	36.928(1.695)***	21.785
Age	0.012 (0.054)	0.225
SES	-0.409(0.321)	-1.275
Cleanliness and Clutter	0.081(0.968)	0.084
Basic Services	7.79(1.597)***	4.877
Hazards	-2.262(1.6)	-1.410
Structural Quality	3.099(1.119)**	2.770
Crowding	-1.332(1.525)	-0.873

Note: B = unstandardized regression coefficient; SE = standard error; t = t-statistic; housing stress model: $R^2 = 0.309$ (adjusted $R^2 = 0.27$), $F(7, 124) = 7.91$, $p < 0.001$.
* $p < .05$; **$p < .01$; ***$p < .001$
